# Dairy Product, Calcium Intake and Lung Cancer Risk: A Systematic Review with Meta-Analysis

**DOI:** 10.1038/srep20624

**Published:** 2016-02-15

**Authors:** Yang Yang, Xu Wang, Qinghua Yao, Liqiang Qin, Chao Xu

**Affiliations:** 1 Department of Radiation Therapy, Zhejiang Cancer Hospital, Hangzhou, China; 2 Zhejiang Key Laboratory of Radiation Oncology, Zhejiang Cancer Hospital, Hangzhou, China; 3 Department of Radiology, Zhejiang Cancer Hospital, Hangzhou, China; 4 Department of Integrated Traditional Chinese and Western Medicine, Zhejiang Cancer Hospital; Key Laboratory of Integrated Traditional Chinese and Western Medicine Hangzhou, Hangzhou, China; 5 Department of Nutrition and Food Hygiene, School of Public Health, Soochow University, Suzhou, China

## Abstract

The effects of dairy products on human health have been studied for years. However, the relationship between dairy products as well as calcium intake and the risk of lung cancer is still inconclusive. A total of 32 studies regarding this association were identified from the PubMed and Web of Science databases through April 1, 2015, including 12 cohort studies and 20 case-control studies. After pooling the results of individual studies, the summary RRs (relative risks) of lung cancer for the highest versus lowest intake were 1.05 (95%CI: 0.84–1.31) and 1.08 (95%CI: 0.80–1.46) for total dairy products and milk, respectively. The results on the consumption of cheese, yogurt and low-fat milk were also negative, and the RRs for total and dietary calcium intakes were 0.99 (95%CI: 0.70–1.38) and 0.85 (95%CI: 0.63–1.13), respectively. After stratifying by potential confounders, the results remained consistent in most subgroup analyses. Our study indicates that intake of dairy products or calcium was not statistically associated with the risk of lung cancer. This negative finding provides a conclusive answer to the disease association issue based on current evidence, and suggests that further efforts should be made to find other nutritional risk factors for lung cancer.

Important components of our diet, dairy products provide a variety of nutrients for humankind, and their effects on human health have been the target of research investigations for years. Milk was once believed to be quite healthy and was recommended strongly in dietary guidelines worldwide. However, concerns regarding the side effects of dairy products on human health have also existed for some time, a recent large cohort study reported that higher milk intake even increased the incidence of fracture and overall mortality, which aroused an intense debate regarding the consumption of milk products^1^.

Considering the diverse effects of dairy products, the connection between dairy intake and specific diseases, especially cancer is a topic worth studying. Previous studies have reported inconsistent results with different cancers. For instance, studies showed that milk and calcium intake might protect against colorectal cancer^2^, but was associated with increased risk of prostate^3^ and ovarian cancers^4^, while no significant association was observed for pancreatic^5^, gastric^6^ and bladder cancers^7^. An important element in milk, calcium plays a crucial role in the biological effects of dairy products and has been found to be associated with the risk for certain malignant tumors^2^,^8^.

As one of most common cancers in both the males and females, lung cancer is the leading cause of cancer-related death worldwide^9^. The primary risk factor for lung cancer is smoking, and accounts for ~80% of the cases in men and ~50% in women. Other risk factors associated with lung cancer include air pollution, cooking fumes, radon and asbestos exposure^10^,^11^. Growing evidence suggests that nutritional factors possibly may play a role in lung cancer development^12^,^13^. Over the past few decades, there have been many studies focused on the association between dairy consumption and lung cancer risk, however, the relationship is still unclear, as the evidence from these studies is scattered and unconvincing. Along with the arrival of the era of evidence-based medicine, it is necessary to collect previous data and summarize the results to make a quantitative conclusion. Therefore, we conducted a meta-analysis of observational studies to clarify the association between the intake of dairy products and calcium with lung cancer risk.

## Methods

### Search strategy

We searched related publications in the PubMed and Web of Science databases through April 1, 2015, with search key words including ‘diet’ or ‘foods’ or ‘dairy’ or ‘milk or ‘cheese’ or ‘yogurt’ or ‘calcium’ combined with ‘lung cancer’ or ‘lung carcinoma’. The reference lists of selected articles were also reviewed to locate additional studies. We restricted our literature search to full-length papers in English.

### Selection Criteria

Studies were eligible for the meta-analysis if they met the following criteria: 1) case-control or cohort design; 2) exposure of interest was dairy consumption (including total dairy, milk, cheese, yogurt and other dairy products) or calcium intake; 3) outcome was lung cancer incidence or mortality; and 4) the estimates of relative risk (or hazard ratio or odds ratios) with corresponding 95% CIs were available. When several studies overlapped or were reported repeatedly, the most recent study with the longest follow-up was included in our analysis.

### Data extraction and quality assessment

We extracted the following data from each original study: author’s name, publication date, location, gender, study design, number of cases, sample size, dietary assessment, case ascertainment, exposures of interest, comparisons, ORs (odds ratios) or RRs (relative risks) with their 95% CIs, and the adjusted variables. We also evaluated the quality of each study according to the Newcastle-Ottawa scale, which is primarily applied to observational studies in systematic reviews^14^. In short, each study was assessed on the following three perspectives: selection (4 points), comparability (2 points), and exposure or outcome (3 points). Thus, the highest score was 9 points, scores of 0–6 and 7–9 were considered as low and high quality, respectively. Two investigators independently extracted all data, and a third investigator settled the differences.

### Statistical methods

We extracted the most fully adjusted relative risks with their 95% CIs (the highest vs the lowest category) from each study, and pooled their logarithms to obtain the summary estimates^15^. We examined the heterogeneity among studies using the Q and *I*^2^ statistics, when the P value for the Q statistic was <0.1 or the *I*^2^ was >50%, the studies were considered to be substantially heterogeneous^16^. In this case, the random-effects model was employed to pool the RRs^17^, otherwise, the fixed-effects model was used^18^.

Considering the heterogeneity across studies, we conducted sub-group analyses by study characteristics, and performed meta-regression analyses to investigate the potential sources of heterogeneity. To assess the impact of specific study on the overall results, we carried out sensitivity analyses by recalculating the pooled estimates after removal of one study each time. We evaluated publication bias with the use of the Egger test and Begg’s rank correlation method, *P* < 0.1 indicated a statistical significance. If publication bias was present, we employed ‘trim and fill’ methods to adjust for the bias. We performed all statistical analyses using STATA 12.0 (StataCorp, College Station, TX, USA), and all tests were two-sided.

## Results

### Study selection and characteristics

A total of 26 studies focused on the association between dairy consumption and lung cancer risk were identified, and an additional 6 studies that reported the calcium intake and lung cancer risk were found, including 12 cohort studies and 20 case-control studies. Among them, 12 studies were conducted in Europe, 12 in the Americas, 7 in Asia, and 1 in the South Pacific. The sample size ranged from 159 to 492, 810, with a sum 765, 752 for dairy products and 581, 187 for calcium intake, and the number of lung cancer cases ranged from 56 to 4, 278. Notably, six publications^19^,^20^,^21^,^22^,^23^,^24^ on dairy products and one publication^8^ on calcium intake were excluded, because of multiple reports from the same population data, however, some data in these publications were obtained for sub-group analyses.

**Figure 1:**
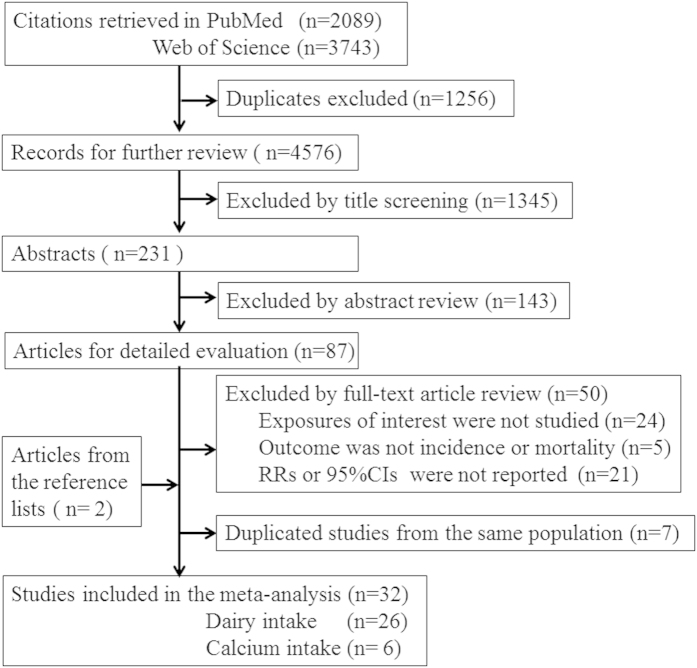
Flow chart of literature search in the meta-analysis.

The quality scores ranged from 4 to 9 with a mean of 6.52, among these studies, 16 were recognized as high quality. In most studies, the adjusted confounding variables covered age, gender and smoking status, however, total energy intake, body mass index and consumption of other foods were not common, especially in the low-quality studies. The study selection process is summarized in Fig. 1, the main characteristics and quality scores of included studies are listed in Supplemental Tables S1 and S2.

### Total dairy

**Figure 2:**
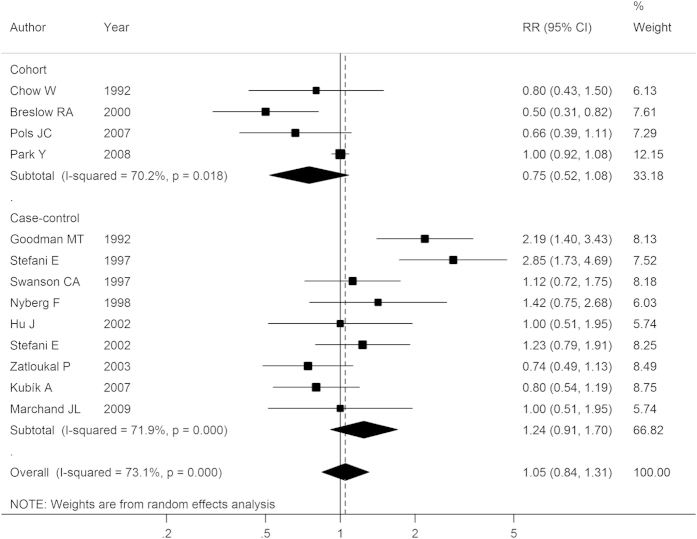
Forest plot of total dairy intake and lung cancer risk for high versus low consumption. RR, relative risk; CI, confidence interval.

Nine case-control studies and 4 cohort studies reported the association between total dairy consumption and lung cancer risk. After pooling all results, the summary RR was 1.05 (95% CI: 0.84–1.31), as shown in Fig. 2. In subgroup analyses, the results were 0.75 (0.52–1.08) and 1.24 (0.91–1.70) in cohort studies and case-control studies, respectively. After stratifying by geographic area, combined analysis of studies in America gave an RR of 1.17 (95% CI: 0.86–1.61), and studies of Europeans also showed no significant association between total dairy intake and lung cancer risk (RR: 0.81, 95% CI: 0.64–1.03). Stratified analyses by other confounders, such as gender, quality and smoking status also yielded similar negative results, indicating the stability of the pooled RRs (Table 1 and Supplementary Fig. 1). Exceptionally, the analysis of three studies^25^,^26^,^27^ investigating the mortality of lung cancer found a significantly inverse association (RR: 0.62, 95% CI: 0.45–0.84), possibly due to a small number of studies included.

A high heterogeneity was observed among all the studies (*I*^2^ = 44.0%, *P*_-heterogeneity_ = 0.04). However, univariate meta-regression analysis indicated that none of the confounders, including study design, location, quality, publication year and number of cases were significantly associated with the heterogeneity. We also conducted a sensitivity analysis in which one study was omitted each time sequentially, and the recalculated RRs ranged from 0.97 (95% CI: 0.80–1.17) to 1.08 (95% CI: 0.87–1.37), indicating that no individual study influenced the overall result significantly. No evidence of publication bias was indicated by Egger’s test (*P* = 0.62) or from Begg’s test (*P* = 0.76).

### Milk

**Figure 3:**
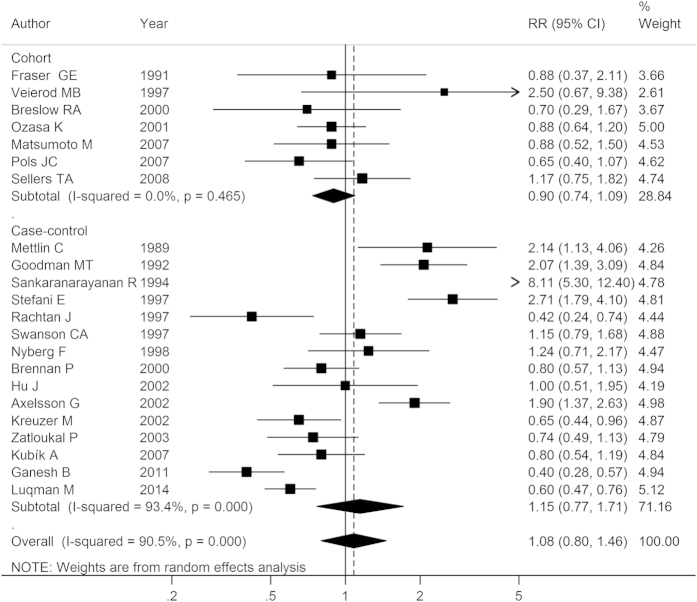
Forest plot of milk intake and lung cancer risk for high versus low consumption. RR, relative risk; CI, confidence interval.

The association between milk consumption and lung cancer risk was examined in 7 cohort studies and 15 case-control studies. Combined analysis by random-effects model showed that the summary RR for the highest versus lowest intake of milk was 1.08 (95% CI: 0.80–1.46), with a high heterogeneity (*I*^2^ = 90.50%, *P-*_heterogeneity_ < 0.01; Fig. 3). The RRs were 0.90 (95% CI: 0.74–1.09) for cohort studies, and 1.15 (95% CI: 0.77–1.71) for case-control studies, respectively. We further performed sub-group analysis stratified by potential confounders, the results were also negative in the majority of cases (Table 1). However, the sub-group analysis of studies in America obtained a positive link between milk intake and lung cancer risk (RR: 1.43, 95% CI: 1.03–1.97), and a negative relationship was observed among the studies that did not adjust for smoking (RR: 0.67, 95% CI: 0.57–0.79) (Supplementary Fig. 5).

To explore the source of heterogeneity, meta-regression analysis was conducted, and the result suggested that only ‘publication year’ was associated with heterogeneity significantly (*P* < 0.05), and the covariate could explain 35.61% of the variance across studies. Multivariate meta-regression analysis with other covariates also confirmed this finding (*P* = 0.012). Sensitivity analysis by removing one study each time demonstrated that the pooled results remained unchanged. We found no significant publication bias, either from Egger’s test (*P* = 0.30) or from Begg’s test (*P* = 0.41).

### Cheese, yogurt and low-fat milk

**Figure 4:**
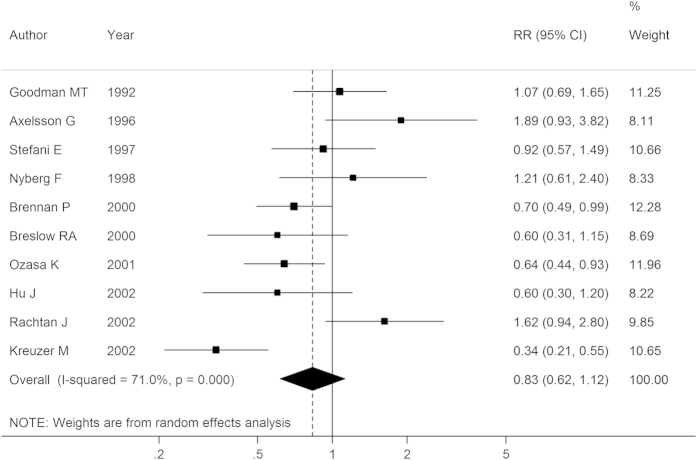
Forest plot of cheese intake and lung cancer risk for high versus low consumption. RR, relative risk; CI, confidence interval.

Ten studies reported the association between cheese intake and lung cancer risk, and the summary RR was 0.83 (95% CI: 0.62–1.12) after combining all the results, with substantial heterogeneity (*I*^2^ = 71.00%, *P-*_heterogeneity_ < 0.01; Fig. 4). Further subgroup or sensitivity analyses did not change the non-significant relationship between cheese intake and lung cancer risk, except for the results in cohort studies (RR: 0.63, 95% CI: 0.46–0.87) or studies that adopted mortality as outcome or that did not adjust for smoking, nevertheless, the numbers of included studies were all quite small (n = 2, 2, 3, respectively). Both Egger’s test (*P* = 0.36) and Begg’s test (*P* = 0.47) did not show any publication bias from the included studies.

Our analysis of five studies on the association of yogurt (or sour milk) intake and lung cancer risk found an RR of 0.88 (95% CI: 0.62–1.25). There was also no significant association (RR: 0.98, 95% CI: 0.69–1.41) between intake of low-fat milk (or skim milk) and lung cancer risk after pooling the results of three studies^26^,^28^,^29^, and no substantial heterogeneity was found among the studies (*I*^2^ = 0.00%, *P-*_heterogeneity_ = 0.43). We did not carry out further sub-group analyses because the included studies were few. No publication bias was found for yogurt (Egger’s test: *P* = 0.63, Begg’s test: *P* = 0.81), or for low-fat milk (Egger’s test: *P* = 0.12, Begg’s test: *P* = 0.30).

### Calcium

Different dietary exposures were reported in the included studies, since calcium is available both from foods and supplements. Six studies^30^,^31^,^32^,^33^,^34^,^35^ provided the data on the association between dietary calcium and lung cancer risk, and the pooled analysis showed the summary RR was 0.85 (95% CI: 0.63–1.13, *I*^2^ = 81.40%, *P-*_heterogeneity_ < 0.01) using a random-effects model. Pooled analysis of only 3 studies^30^,^31^,^32^ on the association between total calcium intake (both diet and supplement) and lung cancer risk gave an RR of 0.99 (95% CI: 0.70–1.38, *I*^2^ = 75.00%, *P-*_heterogeneity_ = 0.02). There were not enough data on the particular calcium source from dairy or supplements to analyze. Further sub-group analyses were not carried out, and the publication bias was not evaluated, due to the limited number of included studies.

## Discussion

In this meta-analysis, we found that the intake of dairy products, including total dairy, milk, cheese, yogurt, or low-fat milk, as well as calcium were not significantly associated with lung cancer risk. The association remained unchanged when stratified by study design, gender, geographic area, quality and smoking status. Our results are consistent with the findings from the largest cohort study (NIH-AARP Diet and Health Study)^8^, and this null association was also found in many other types of cancer, such as gastric^6^, bladder^7^ and pancreatic cancers^5^. Our study involved a large sample size, covered a long time span from 1989 to 2012, and thus provided a conclusive answer to the association issue between dairy consumption and lung cancer risk.

Dairy products are nutrient-rich foods, which contain carbohydrates, fatty acids, proteins, vitamins, minerals and small bioactive molecules. However, the effects of dairy products on human health are just as complex as their constituents, and the role of dairy products in cancer development remains controversial. On one hand, some studies showed that dairy might be beneficial for cancer prevention as it contains various minerals and vitamins^36^. The most remarkable example is the protective effect of vitamin D and calcium intake on colorectal cancer^37^,^38^. However, in our meta-analysis, calcium intake appears not to be associated with a lower risk of lung cancer. In addition, we analyzed the conflicting results of several studies that reported the association of vitamin D intake and lung cancer risk^39^,^40^,^41^. The pooled RR for highest versus lowest supplements of vitamin D was 0.97 (95% CI: 0.80–1.18), with no heterogeneity observed (*I*^2^ = 0.00%, *P-*_heterogeneity_ = 0.43), indicating that vitamin D supplements are not likely to be helpful for lung cancer prevention. The consistency of the results from different types of dairy products, calcium and vitamin D intake, supported the null association to some degree.

On the other hand, some others argue that high content of lactose, D-galactose in milk might promote oxidative stress, which will induce aging, chronic inflammation and other injuries to the organism. Consistent with this view, Ji *et al* recently performed a large cohort study that demonstrated that individuals with lactose intolerance, who consumed less milk or dairy products had a lower risk of lung cancer^42^. Additionally, increases in levels of saturated fat, exogenous DNA agents^43^, several bioactive components like IGF-1 (insulin-like growth factor-1) and hormone metabolites in milk are also suggested to contribute to the development of many types of cancer^44^. In contrast, a previous meta-analysis, showed that circulating levels of IGF-1 in plasma, was not associated with lung cancer^45^. In fact, studies on the influence of specific components in dairy products on lung cancer performed *in vitro* or in animal models are quite few, which might be explained by the fact that their relationship is not close, since negative results often remain to be unpublished.

As a systematic quantitative method, meta-analysis is useful to reveal those possible associations that are not so obvious in individual studies. It also allows more sub-group analyses to be stratified by confounders, thus it enables a reliable conclusion. However, several shortcomings should also be noted in our analysis. First, to increase the sample size, both case-control and cohort studies were included in the meta-analysis, as a known fact, retrospective studies are susceptible to recall and selection bias. Indeed, there are some differences in the results of case-control studies and cohort studies, however, the differences were not statistically significant, except that the result of cheese intake in cohort studies was inversely significant (RR: 0.63, 95% CI: 0.46–0.87), which might be explained by the small number of studies included.

Secondly, since different exposure ranges and dietary assessment were used in original studies, we did not perform dose-responses effect analyses, and the lack of association also reduced the need for performing this analysis. The limitations described above contributed to high heterogeneity across studies, other factors like baseline characteristics of the population, ascertainment of lung cancer cases and different adjusted confounders also influenced the results. Specifically, high heterogeneity was found in our meta-analysis, especially in lower-quality or case-control studies, in according with our expectations. Meta-regression analyses were used to explore the sources of heterogeneity, however, significant factors were not easily located. In particular, lung cancer is a highly heterogeneous disease with various genetic backgrounds, which may have contributed to increased discrepancy between studies.

In addition, in our sub-group analyses, because of different dietary patterns between Western and Eastern countries, the results from Asia were relatively few, which weakened the statistical power. However, we did not observe much differences in the results between Asian and Western countries. Another problem was the confounder of smoking, as shown in Table 1, as there were indeed significant differences between the results of the studies whether they adjusted for smoking or not in the categories of milk and cheese intakes, implying the confounding effect of smoking on the results. Yet, the data stratified by smoking status were not commonly provided in the original studies, unless the results were a bit abnormal, which increased the artificial bias and limited our sub-group analyses. Thus, we only roughly combined the results of both total dairy products and milk consumption in different smoking groups, and found that the results were non-significantly positive in smokers and non-smokers.

Lastly, both the incidence and mortality of lung cancer were included in our study, and interestingly, subgroup analyses stratified by outcome showed that there were significant differences between the two groups in the categories of dairy and cheese intake (as shown in Table 1). Dairy and cheese intake was inversely associated with lung cancer mortality, this might be explained by the possibility that some components in dairy products are beneficial for the survival of lung cancer. In fact, some studies reported that intake of vitamin D was associated with improved survival in early stage lung cancer patients^46^,^47^. However, these mortality studies are limited, and the pooled RR was not significant in the category of milk when more studies were included. As such, additional studies are needed to investigate the association between dairy product intake and lung cancer mortality and survival.

Hence, based on both epidemiological data and biological evidence, there is no apparent association between the intake of dairy products or calcium and the risk of lung cancer. Future efforts should be focused on seeking other nutritional risk factors for lung cancer. Furthermore, there were indeed some concerns regarding the influence of dairy products on the incidence of cancer, especially prostate and ovarian cancers. To avoid the harmful effects of dairy products, more advanced technology in milk processing is urgently needed, which might reduce lactose content, bioactive components and other detrimental ingredients found in dairy products.

## Conclusion

In conclusion, our meta-analysis demonstrates that dairy product or calcium intake is not significantly associated with lung cancer risk. Although it is a negative finding, it provides a conclusive answer to the issue, and reveals an intrinsic connection based on the current evidence.

## Additional Information

**How to cite this article**: Yang, Y. *et al.* Dairy Product, Calcium Intake and Lung Cancer Risk: A Systematic Review with Meta-analysis. *Sci. Rep.* **6**, 20624; doi: 10.1038/srep20624 (2016).

## Supplementary Material

Supplementary Information

## Figures and Tables

**Table 1:** Subgroup analyses of dairy product and calcium intakes and lung cancer risk, high vs low intake.

Subgroups		*N*	RR (95% CI)	Heterogeneity			*P*_-heterogeneity_	*P*-_interaction_
				*P* value	Q statistic	*I*^*2*^		
Total dairy		13	1.05 (0.84–1.31)	0.67	44.67	73.10%	<0.01	
Design	Case-control	9	1.24 (0.91–1.70)	0.17	28.47	71.90%	<0.01	
	Cohort	4	0.75 (0.52–1.08)	0.12	44.67	70.20%	0.018	0.07
Ethnicity	America	8	1.17 (0.86–1.61)	0.32	37.14	81.20%	<0.01	
	Europe	4	0.81 (0.64–1.03)	0.14	3.77	20.40%	0.29	0.41
Outcome	Incidence	10	1.20 (0.94–1.52)	0.14	33.04	72.80%	<0.01	
	Mortality	3	0.62 (0.45–0.84)	<0.01	1.43	0.00%	0.489	0.04
Quality	Low	6	1.30 (0.83–2.05)	0.26	24.60	79.70%	<0.01	
	High	7	0.89 (0.71–1.10)	0.28	12.35	51.40%	0.06	0.14
Sex	Male	5	1.46 (0.95–2.25)	0.08	24.46	83.60%	<0.01	
	Female	6	0.92 (0.82–1.02)	0.13	4.23	0.00%	0.52	0.37
No. of cases	≤300		0.88 (0.66–1.18)	0.39	11.06	45.70%	0.09	
	>300		1.23 (0.87–1.74)	0.23	31.48	84.10%	<0.01	0.22
Smoking†	Smokers	4	1.32 (0.65–2.68)	0.44	21.90	86.30%	<0.01	
	Non-smokers	4	1.55 (0.98–2.45)	0.06	4.75	36.80%	0.19	0.93
Adjustment for confounders								
Smoking	Yes	11	1.09 (0.86–1.39)	0.46	42.00	76.20%	<0.01	
	No	2	0.77 (0.51–1.16)	0.22	0.93	0.00%	0.34	0.44
Energy intake	Yes	6	1.16 (0.85–1.60)	0.34	20.11	75.10%	<0.01	
	No	7	0.95 (0.65–1.40)	0.80	23.83	74.80%	<0.01	0.45
Milk		22	1.08 (0.80–1.46)	0.61	220.15	90.50%	<0.01	
Design	case-control	15	1.15 (0.77–1.71)	0.50	212.73	93.40%	<0.01	
	Cohort	7	0.90 (0.74–1.09)	0.28	5.64	0.00%	0.46	0.55
Location	America	8	1.43 (1.03–1.97)	0.03	20.46	65.80%	<0.01	
	Europe	9	0.87 (0.63–1.21)	0.87	135.07	97.00%	<0.01	
	Asia	5	1.08 (0.44–2.65)	0.40	36.61	78.20%	<0.01	0.47
Outcome	Incidence	18	1.16 (0.81–1.67)	0.41	214.78	92.10%	<0.01	
	Mortality	4	0.81 (0.65–1.02)	0.08	1.22	0.00%	<0.01	0.32
Quality	Low	13	1.08 (0.80–1.46)	0.56	210.79	94.30%	<0.01	
	High	9	0.94 (0.80–1.12)	0.54	8.45	5.40%	0.39	0.61
Sex	Male	6	1.76 (0.78–3.97)	0.18	143.22	96.50%	<0.01	
	Female	10	0.94 (0.73–1.22)	0.65	25.06	64.10%	<0.01	0.13
No. of cases	≤300	11	1.09 (0.62–1.92)	0.77	110.98	91.00%	<0.01	
	>300	11	1.07 (0.75–1.53)	0.69	105.74	90.50%	<0.01	0.99
Adjustment for confounders								
Smoking	Yes	15	1.35 (0.90–2.01)	0.14	163.60	91.40%	<0.01	
	No	7	0.67 (0.57–0.79)	<0.01	6.84	12.30%	0.34	0.03
Energy intake	Yes	5	1.21 (0.75–1.94)	0.79	193.11	91.70%	<0.01	
	No	17	1.05 (0.73–1.51)	0.44	20.83	80.80%	<0.01	0.72
								
Cheese		10	0.83 (0.62–1.12)	0.23	31.04	71.00%		
Design	Case-control	8	0.90 (0.62–1.30)	0.58	28.59	75.50%	<0.01	
	Cohort	2	0.63 (0.46–0.87)	<0.01	0.03	0.00%	0.87	0.38
Location	America	4	0.84 (0.64–1.12)	0.24	3.26	8.10%	0.35	
	Europe	5	0.94 (0.52–1.72)	0.85	26.22	84.70%	<0.01	
	Asia	1	0.64 (0.44–0.93)	0.02	/	/	/	0.49
Outcome	Incidence	8	0.90 (0.62–1.31)	0.58	28.67	75.60%	<0.01	
	Mortality	2	0.63 (0.46–0.87)	<0.01	0.03	00.00%	0.87	0.38
Quality	Low	6	0.78 (0.52–1.19)	0.25	20.83	76.00%	<0.01	
	High	4	0.92 (0.56–1.50)	0.73	9.67	69.00%	0.02	0.67
No. of cases	≤300	5	0.75 (0.41–1.37)	0.35	20.53	80.50%	<0.01	
	>300	5	0.89 (0.66–1.21)	0.47	9.53	58.00%	0.05	0.52
Adjustment for confounders								
Smoking	Yes	7	1.01 (0.74–1.38)	0.93	14.49	58.60%	0.03	
	No	3	0.53 (0.33–0.85)	<0.01	5.75	65.20%	0.06	0.05
Energy intake	Yes	2	0.80 (0.54–1.19)	0.27	0.99	0.00%	<0.01	
	No	8	0.85 (0.59–1.23)	0.39	30.17	76.80%	0.32	0.26
Yogurt		5	0.88 (0.62–1.25)	0.48	9.93	59.70%	0.04	
low-fat milk		3	0.98 (0.69–1.41)	0.93	1.68	0.00%	0.43	
Calcium								
	Total	3	0.99 (0.70–1.38)	0.94	8.00	75.00%	0.02	
	Dietary	5	0.85 (0.63–1.13)	0.26	26.87	81.40%	<0.01	/

Note: For subgroup analysis stratified by smoking status, the data on the categories of dairy and milk intakes were both included, since only a small number of original studies reported the results, the forest plot was shown in Supplementary Fig. S1.
