# Supplementary material for: Dairy Product, Calcium Intake and Lung Cancer Risk: A Systematic Review with Meta-Analysis
Source: Sci Rep. 2016 Feb 15;6:20624. doi: 10.1038/srep20624 (PMC4753428; doi:10.1038/srep20624)

Supplementary Information

Dairy Product, Calcium Intakes and Lung Cancer Risk: A Systematic Review with Meta-analysis

Yang Yang, Xu Wang, Qinghua Yao, Linqiang Qin

Table S1 Characteristics of studies included in the meta-analysis on the dairy product intake and lung cancer risk.

Author	Year	Location	Duration	Design	Sex	No. of cases	Size/ control	Dietary assessment	Diagnosis method	Exposures	Comparisons	RRs (Highest to lowest)	Adjusted variables	Quality
Mettlin C[25]	1989	USA	1982-1987	Case-control/h	Both	569	569	FFQ-45 items/I	Histology	Whole milk<br>2% milk<br>Skim milk	3 or more/day vs never<br>3 or more/day vs never<br>3 or more/day vs never	2.14(1.13-4.07)<br>0.52(0.30-0.90)<br>0.68(0.28-1.67)	Sex, smoking history, beta-carotene intake index and education level	5
Fraser G[26]	1991	USA	1977-1982	Cohort	Both	61	34198	FFQ-51 items/S	Histology	Milk	>7 ts/w vs <1 ts/w	0.88(0.37-2.12)	Age, sex, and smoking history	7
Chow W[27]	1992	USA	1966-1986	Cohort	Male	219	17633	Questionnaire	Death certificates	Dairy	>142 ts/m vs <46 ts/m	0.80(0.4-1.4)	Age, smoking status, and industry/occupation	6
Goodman M[28]	1992	USA	1983-1985	Case–control/p	Both	326	865	FFQ-130 items/I	Histology	Dairy foods<br>whole milk<br>low-fat milk<br>Cheese	Quartile 4 vs Quartile 1<br>Quartile 4 vs Quartile 1<br>Quartile 4 vs Quartile 1<br>Quartile 4 vs Quartile 1	2.19(1.40-3.43)<br>2.07(1.39-3.09)<br>1.02(0.641.64)<br>1.07(0.69-1.64)	Age, ethnicity, smoking status, pack-years of cigarette use and beta-carotene intake	6
Sankaranarayanan[29]	1994	India	1990-1994	Case–control/h	Male	281	1281	FFQ-45items/I	Registry	Milk,<br>Buttermilk	ever vs never<br>ever vs never	8.11(5.30-12.3)<br>2.70(1.86-3.93)	Age, education, religion and smoking	6
Axelsson G[30]	1996	Sweden	1989-1993	Case–control/p	Both	308	504	FFQ-130 items/I	Registry	Milk<br>Sour milk<br>Cheese	several ts/d vs ≤2 ts/m<br>daily vs ≤ 2 ts/m<br>several ts/d vs ≤ 2 ts/m	1.73(1.00-3.01)<br>0.98(0.65-1.48)<br>1.89(0.94-3.80)	Number of cigarettes/day, number of years smoked, marital status, socioeconomic job classification, vegetable class and other fruit or berries	5
Veierod M[31]	1997	Norway	1977-1991	Cohort	Both	153	51452	FFQ-80 items	Registry	Milk	whole milk vs no milk	2.5 (0.71-10)	Smoking status, gender, age at screening, and attained age	8
Stefani E[32]	1997	Uruguay	NR	Case-control/h	Male	377	377	FFQ-64 items	NR	Dairy<br>Whole milk<br>Cheese	Quartile 4 vs Quartile 1<br>Tertile 3 vs Tertile 1<br>Tertile 3 vs Tertile 1	2.85(1.73–4.69)<br>2.71(1.80–4.11)<br>0.92(0.57–1.49)	age , residence, education, family history of lung cancer, body-mass index , alpha-carotene , tobacco smoking and total energy intake	6
Rachtan J[33]	1997	Poland	1991-1994	Case–control/h	Female	118	141	FFQ-17 items/I	Histology	Milk	≥2 ts/w vs Rarely	0.42 (0.24–0.75)	Age	5
Swanson CA[34]	1997	USA	1993-1994	Case-control/p	Female	587	624	FFQ-100 items/I	Registry	Dairy<br>Milk	23.8+ vs <7.0 ts/w<br>16.8+ vs < 6.0 ts/w	1.12(0.70-1.70)<br>1.15 (0.8-1.70)	Age and total calories, education, pack-years of smoking, body mass index, consumption of vegetables and fruit	7
Nyberg F[35]	1998	Sweden	1989-1995	Case–control/p	Both	124	35	Questionnaire	Histology	Milk<br>yogurt<br>Both above<br>Cheese	>2 glasses/d vs < daily<br>daily vs weekly<br>>2 glasses/ d vs < daily<br>>4 slices/d vs < daily	1.24(0.71–2.17)<br>1.61(0.91–2.85)<br>1.42(0.75-2.68)<br>1.21 (0.61–2.39)	Gender, age , catchment ,occasional smoking; degree of urban residence; years of exposure to risk occupations; ever-exposure status	7
Brennan P[36]	2000	Europe	NR	Case-control/P	Both	506	1045	FFQ/I	NR	Milk<br>Cheese	several ts/w vs <1t//m<br>several ts/w vs <1t/m	0.80(0.60-1.20)<br>0.70 (0.50-1.00)	Age, sex and center	6
Breslow RA[37]	2000	USA	1987-1995	Cohort	Both	158	19846	FFQ-59 items /I	Registry	Dairy<br>Cheese<br>Whole milk<br>2% milk<br>Skim milk<br>1% milk	Quartile 4 vs Quartile 1<br>Quartile 3 vs Quartile 1<br>Tertile 3 vs Tertile 1<br>Tertile 3 vs Tertile 1<br>Tertile 3 vs Tertile 1	0.50(0.30-0.80)<br>0.60(0.30-1.10)<br>0.70(0.30-1.70)<br>0.50(0.20-1.40)<br>0.80 (0.40-1.60)	Age, gender and smoking	7
Ozasa K[38]	2001	Japan	1988-1997	Cohort	Both	572	98248	FFQ-32 items/S	Death certificates	Milk<br>Yogurt<br>Cheese	daily vs scarcely any<br>3-4/w+ vs scarcely any<br>3-4/w+ vs scarcely any	0.87(0.69-1.11)<br>0.81(0.58-1.14)<br>0.64(0.44-0.92)	Age, Parents' History of Lung Cancer, Smoking Status, Smoking Index and Time since Quitting Smoking	8
Hu J[39]	2002	Canada	1994-1997	Case–control/p	Female	161	484	Questionnaire	Histology	Milk<br>Cheese	Quartile 4 vs Quartile 1<br>Quartile 3 vs Quartile 1	1.00(0.50-1.90)<br>0.60 (0.30–1.20)	Age groups, province, education, social class and total energy intake	6
Rachtan J[40]	2002	Poland	1991-1997	Case–control/h	Female	242	352	Questionnaire	Histology	Cheese	> 3 ts/w vs rarely	1.62(0.94-2.81)	Age, pack years of smoking, passive smoking, consumption	7

													of beer and vodka, siblings with cancer, tuberculosis, place of residence, occupation exposure	
Kreuzer M[41]	2002	Germany	1991-1996	Case-control/p	Female	234	535	FFQ-15 items /I	Histology	Milk	daily vs monthly	0.65(0.44–0.95)	Age and region	6
										Yogurt	daily vs monthly	0.53(0.34–0.81)		
										Cheese	daily vs monthly	0.34(0.21–0.55)		
Zatloukal P[42]	2003	Czech Republic	1998-2002	Case-control/h	Female	366	1624	Questionnaire	Histology	Dairy	daily vs monthly	0.74 (0.48-1.11)	Age, residence, education and pack-years of smoking	5
Kub k A[43]	2007	Czech Republic	1997-2005	Case-control/h	Female	569	2120	Questionnaire	Histology	Dairy	daily or weekly	0.80 (0.54-1.20)	Age, residence and education, smoking	7
Matsumoto M[44]	2007	Japan	1992-2002	Cohort	Both	56	11349	FFQ-30 items	Death certificates	Milk	daily vs not everyday	0.88(0.52-1.51)	Sex and age	6
										Butter	daily vs not everyday	1.66(0.66-4.15)		
										Yogurt	daily vs not everyday	0.95(0.29-3.03)		
Pols J[45]	2007	Britain	1948-2005	Cohort	Both	153	4374	7-d household inventory	Death certificates	Total dairy	Quartile 4 vs Quartile 1	0.66(0.39-1.10)	Age, sex, and energy and fruit intakes	8
										Milk	Quartile 4 vs Quartile 1	0.65 (0.40-1.08)		
Sellers T[46]	2008	USA	1986-2005	Cohort	Female	229	22808	FFQ-milk/S	Registry	Milk	As adult vs never	1.17 (0.75-1.82)	Age , caloric intake, physical activity, BMI, smoking status, HRT, farm resident status, educational status, height, parity/age at first birth, marital status, history of blood transfusions, diabetes mellitus, age at menopause	9
Park Y[8]	2008	USA	1995-2003	Cohort	Both	4278	492810	FFQ-124 items /S	Registry	Dairy	Quintile 5 vs Quintile 1	1.00(0.92-1.08)	race, education, marital status, BMI , family history of cancer, physical activity, and intakes of red meat and total energy, smoking	8
Marchand J[47]	2009	New Caledonia	1993-1995	Case-control/p	Both	134	295	FFQ-89 items/I	Registry	Dairy	Tertile 3 vs Tertile 1	1.00(0.50–1.90)	smoking, age, and ethnicity	7
Takata Y[48]	2013	China	1997-2009	Cohort	Female	428	71267	FFQ/I	Histology	Dairy foods	Any vs none	0.79(0.65–0.96)	Age, total caloric intake, income, occupation, passive smoking, history of asthma and BMI	9
Luqman M[49]	2014	Pakistan	2010-2013	Case-control/h	Both	400	800	Questionnaire	Histology	Milk	Yes vs No	0.60 (0.50-0.80)	None	4

Abbreviations: h: hospital-based; p: population-based; FFQ: food frequency questionnaire; NR: not reported; I: interviewed; S: self-reported or mailed; ts: times; d: day; w: week: m: moth; HRT: hormone replacement therapy; BMI: body mass index.

Table S2 Characteristics of studies included in the meta-analysis on the calcium intake and lung cancer risk.

**Author**	**Year**	**Location**	**Duration**	**Design**	**Sex**	**No. of case**	**Size/ Control**	**Dietary assessment**	**Diagnosis method**	**Exposures**	**Comparisons**	**RRs (Highest to lowest)**	**Adjusted variables**	**Quality**
Hu J [50]	1997	China	1985-1987	Case-control/h	Both	227	227	Questionnaire-	NR	Dietary calcium	Quartile 4 vs Quartile 1	0.80(0.50–1.40)	Cigarettes per day, duration and family income	5
Zhou W[51]	2005	USA	1992-2000	Case-control/h	Both	923	1125	FFQ- 26 items	Histology	Total calcium<br>Dietary calcium	Quintile 5 vs Quintile 1<br>Quintile 5 vs Quintile 1	1.45(1.01-2.08)<br>1.81(1.27-2.56)	Age, sex, smoking status, pack-years of smoking, years since smoking cessation, total energy, and education levels	6
Mar á S[52]	2009	Argentina	NR	Case-control/h	Male	87	153	FFQ	Histology	dietary calcium	>1544 mg/d vs <962 mg/d	0.34(0.14–0.84)	Smoking habit, occupation, BMI and energy intake	5
Mahabir S[53]	2010	USA	1995-2003	Cohort	Both	7052	482875	FFQ-124items/S	Registry	Total calcium<br>Dietary calcium	>1362 mg/d vs≤ 608 mg/d<br>>952 mg/d vs≤535 mg/d	0.97(0.88–1.07)<br>0.92(0.84–1.01)	Age, sex, race, education, cigarette smoke dose, smoke quit, BMI, physical activity, alcohol intake, and food calories, total other minerals.	8
Li K[54]	2011	Germany	1994–1998	Cohort	Both	147	25540	FFQ/S	Histology	Dietary calcium	Quartile 4 vs Quartile 1	0.71(0.41–1.21)	Sex, age at recruitment, educational level, physical activity, BMI, waist-to-hip ratio, smoking category, lifetime alcohol intake, meat/meat product intake, dietary intakes of vitamin D, vitamin K2, and fiber, total energy intake, and regular use of Ca/vitamin D supplements	8
Takata Y[48]	2013	China	1997-2009	Cohort	Female	428	71267	FFQ/I	Histology	Total calcium<br>Dietary calcium<br>Supplements	>578 mg/d vs <321 mg/d<br>>578 mg/d vs <321 mg/d<br>>578 mg/d vs <321 mg/d	0.65(0.42-1.00)<br>0.66(0.48-0.91)<br>0.89(0.72–1.10)	Age, total caloric intake, income, occupation, passive smoking, history of asthma and BMI	9

Abbreviations: h: hospital-based; p: population-based; FFQ: food frequency questionnaire; NR: not reported; I: interviewed; S: self-reported or mailed; ts: times; d: day; w: week: m: moth; BMI: body mass index.

Figure S1 Subgroup analysis of the association between dairy/milk intake and lung cancer risk, stratified by smoking status.

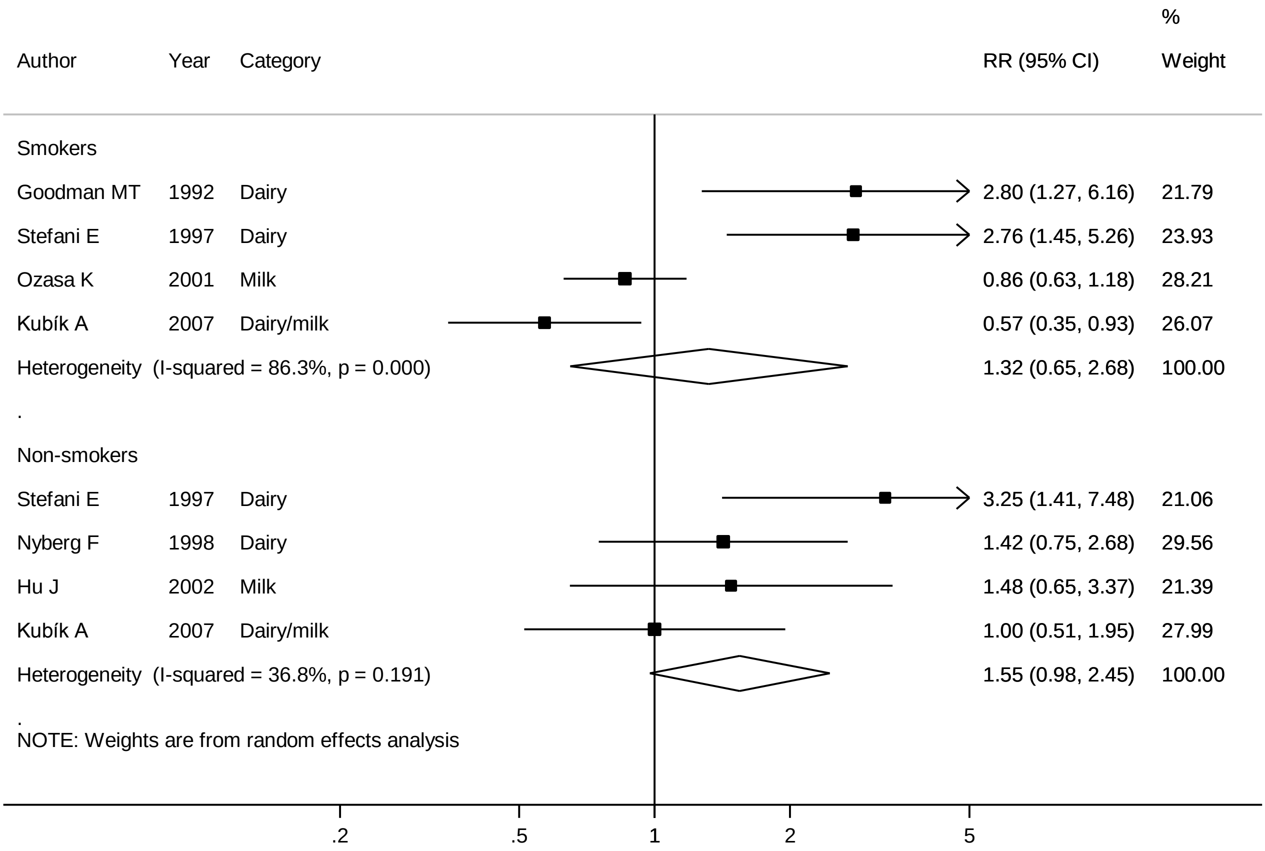

Figure S2 Subgroup analysis of the association between dairy intake and lung cancer risk, stratified by outcome.

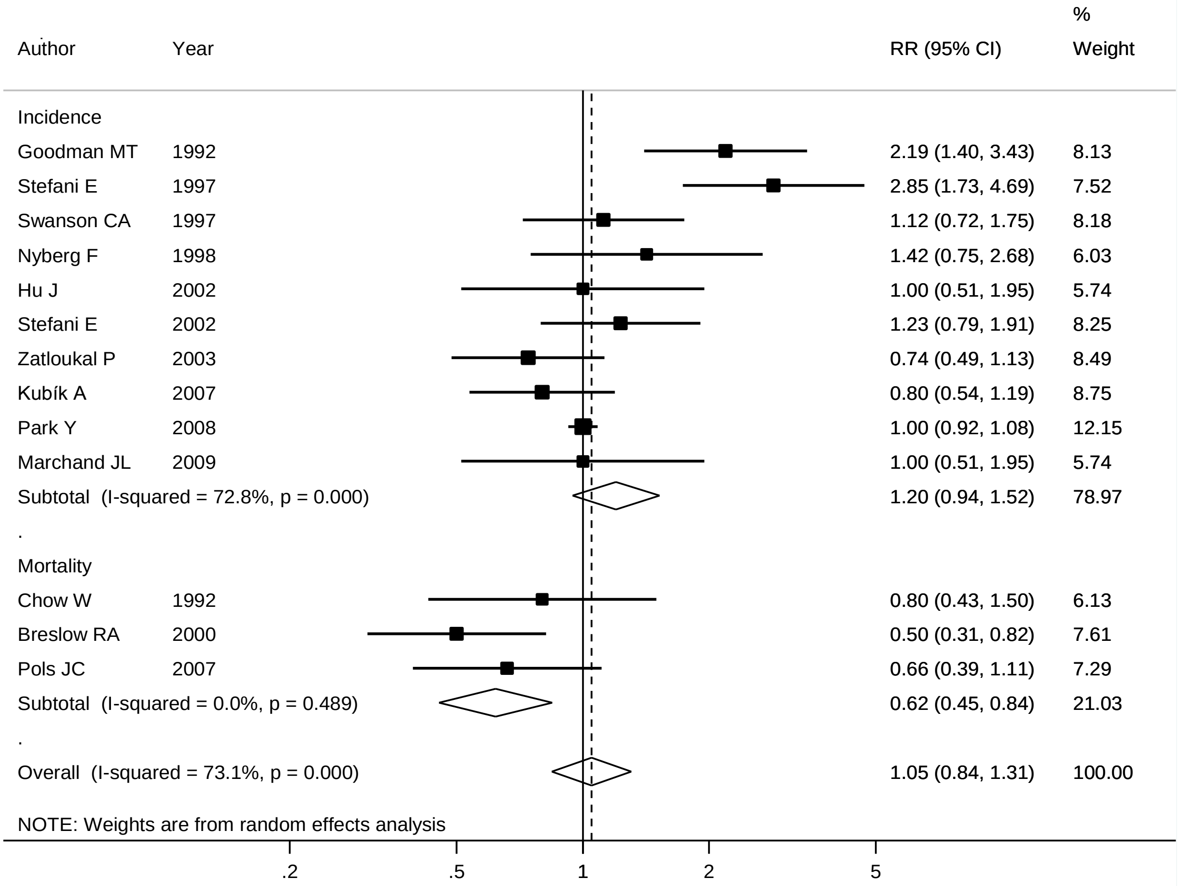

Figure S3 Subgroup analysis of the association between dairy intake and lung cancer risk, stratified by adjustment for smoking status.

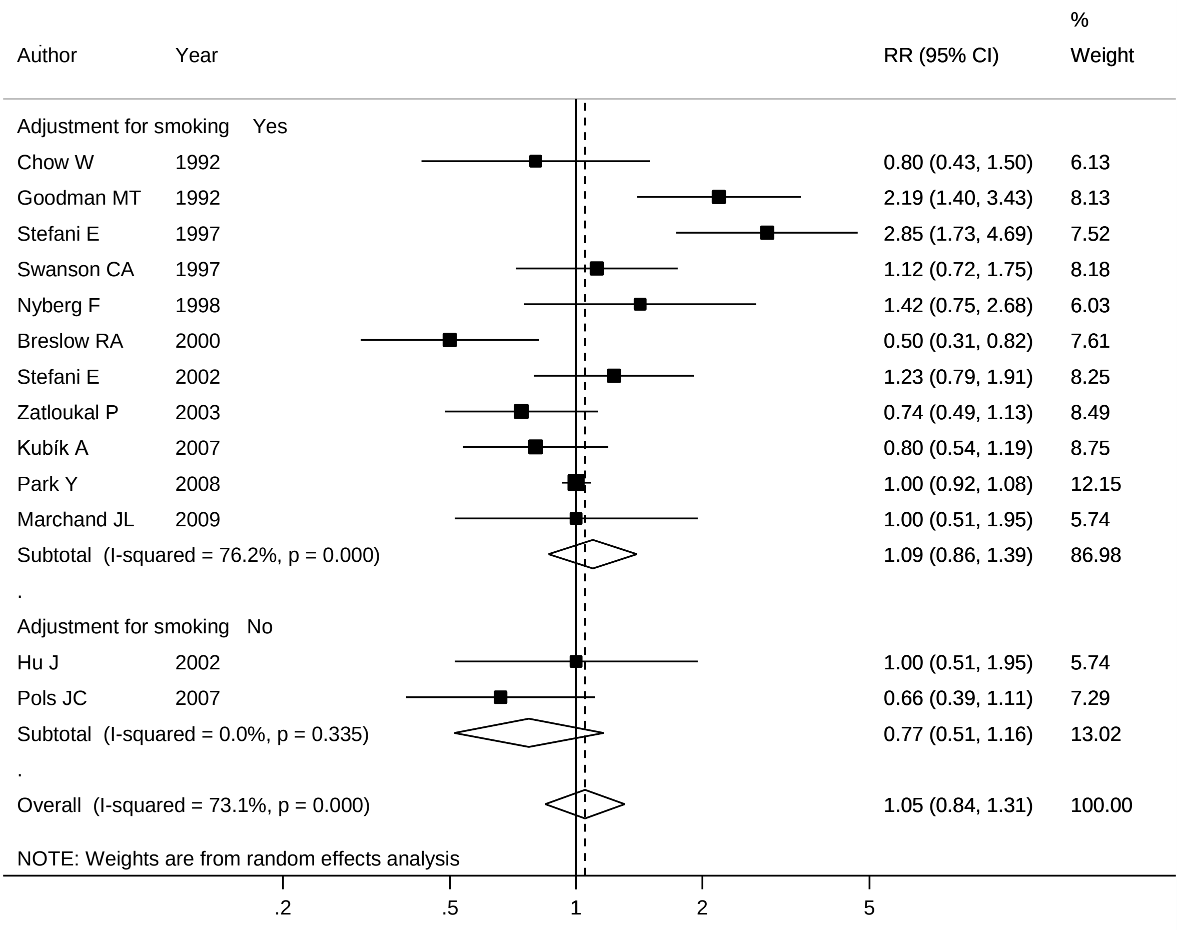

Figure S4 Subgroup analysis of the association between milk intake and lung cancer risk, stratified by outcome.

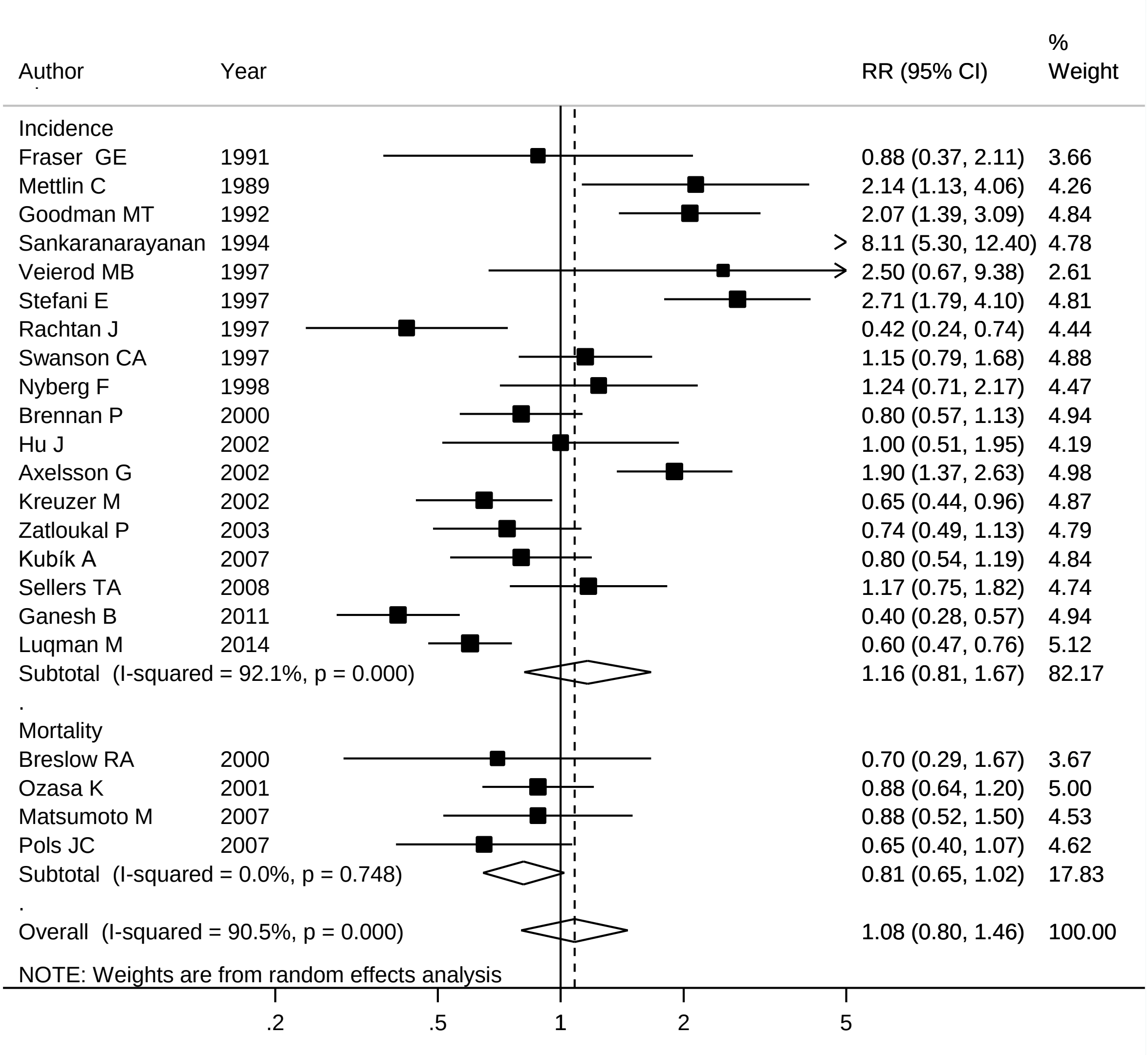

Figure S5 Subgroup analysis of the association between milk intake and lung cancer risk, stratified by adjustment for smoking status.

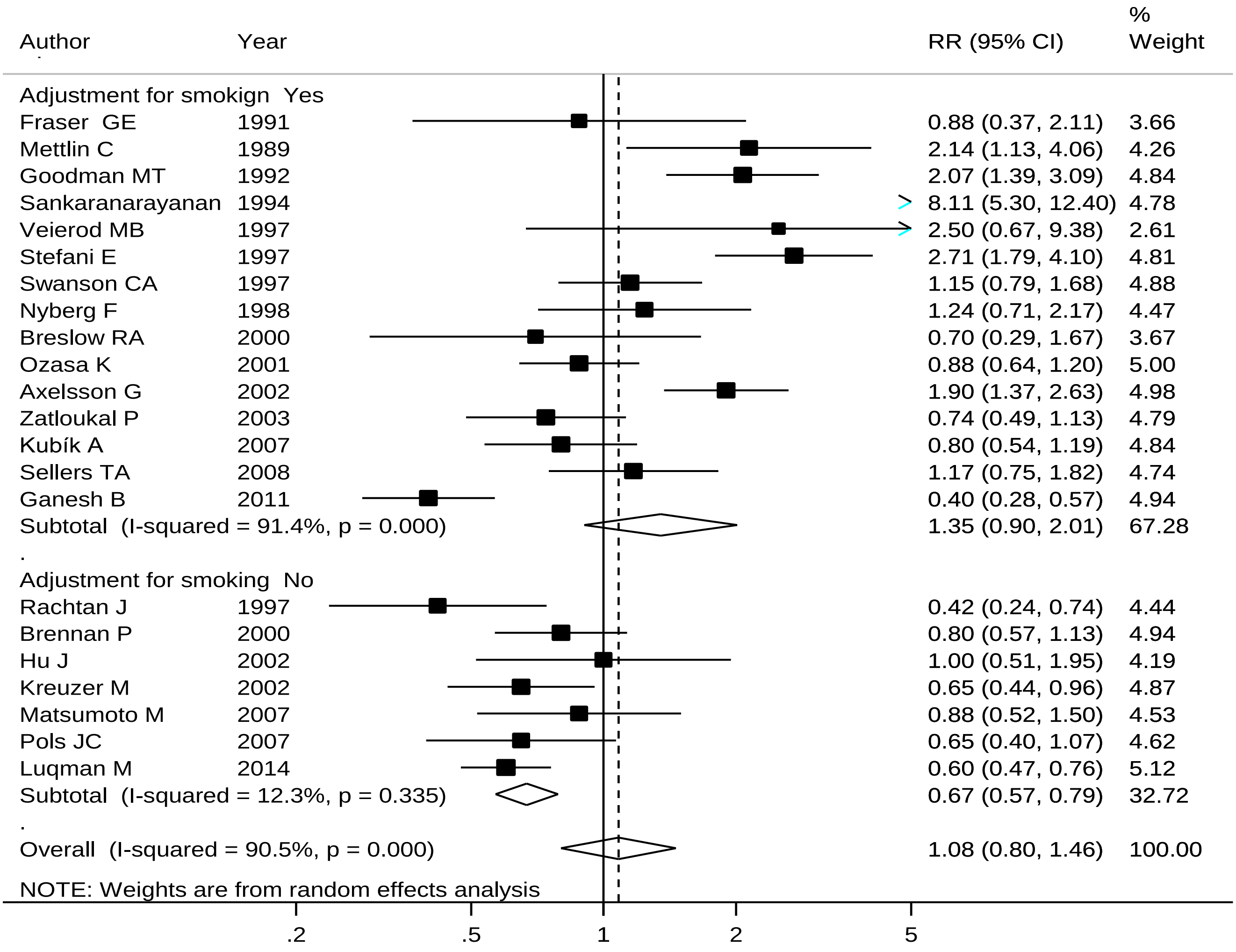

Supplement: Supplementary Information [file srep20624-s1.pdf]
